# Characterization of miR-335-5p and miR-335-3p in human osteoarthritic tissues

**DOI:** 10.1186/s13075-023-03088-6

**Published:** 2023-06-16

**Authors:** Thomas G. Wilson, Madhu Baghel, Navdeep Kaur, Vasilios Moutzouros, Jason Davis, Shabana Amanda Ali

**Affiliations:** 1 Bone and Joint Center, Henry Ford Health, 6135 Woodward Avenue, Detroit, MI 48202 USA; 2 Department of Orthopedic Surgery, Henry Ford Health, Detroit, MI USA; 3 Department of Physiology, Michigan State University, East Lansing, MI USA; 4 Center for Molecular Medicine and Genetics, Wayne State University, Detroit, MI USA

**Keywords:** Osteoarthritis, MicroRNAs, Human tissues, Knee, Hip

## Abstract

**Objective:**

We aimed to characterize the expression patterns, gene targets, and functional effects of miR-335-5p and miR-335-3p among seven primary human knee and hip osteoarthritic tissue types.

**Methods:**

We collected synovial fluid, subchondral bone, articular cartilage, synovium, meniscus/labrum, infrapatellar/acetabular fat, anterior cruciate ligament/ligamentum teres, and vastus medialis oblique/quadratus femoris muscle (*n* = 7–20) from surgical patients with early- or late-stage osteoarthritis (OA) and quantified miR-335-5p and miR-335-3p expression by real-time PCR. Predicted gene targets were measured in knee OA infrapatellar fat following miRNA inhibitor transfection (*n* = 3), and prioritized gene targets were validated following miRNA inhibitor and mimic transfection (*n* = 6). Following pathway analyses, we performed Oil-Red-O staining to assess changes in total lipid content in infrapatellar fat.

**Results:**

Showing a 227-fold increase in knee OA infrapatellar fat (the highest expressing tissue) versus meniscus (the lowest expressing tissue), miR-335-5p was more abundant than miR-335-3p (92-fold increase). MiR-335-5p showed higher expression across knee tissues versus hip tissues, and in late-stage versus early-stage knee OA fat. Exploring candidate genes, *VCAM1* and *MMP13* were identified as putative direct targets of miR-335-5p and miR-335-3p, respectively, showing downregulation with miRNA mimic transfection. Exploring candidate pathways, predicted miR-335-5p gene targets were enriched in a canonical adipogenesis network (*p* = 2.1e − 5). Modulation of miR-335-5p in late-stage knee OA fat showed an inverse relationship to total lipid content.

**Conclusion:**

Our data suggest both miR-335-5p and miR-335-3p regulate gene targets in late-stage knee OA infrapatellar fat, though miR-335-5p appears to be more prominent, with tissue-, joint-, and stage-specific effects.

**Supplementary Information:**

The online version contains supplementary material available at 10.1186/s13075-023-03088-6.

## Introduction

The prevalence of osteoarthritis (OA) is rising with worldwide estimates of knee OA at 364 million and hip OA at 33 million as of 2019 [1]. Now considered a disease of the entire synovial joint, OA affects multiple intra- and extra-articular tissues, yet the most commonly explored tissues remain cartilage, synovium, and subchondral bone [2, 3]. Despite the joint-level and tissue-level differences in OA presentation, disease mechanisms are often not directly compared and contrasted across joints and tissues. This is particularly important for context-dependent epigenetic factors such as microRNAs (miRNAs) — small, non-coding RNAs that function to inhibit gene targets with high specificity based on seed-sequence binding [4–6]. Given the mounting evidence of the key roles miRNAs play in joint health and disease [4], detailed characterization of their function can provide important mechanistic and therapeutic insights for OA.

Following transcription from their host gene, mature miRNAs can be produced from either the 5′ or 3′ arm of precursor molecules, noted in the nomenclature as -5p or -3p, respectively. The biogenesis of mature miRNAs begins following the formation of stem-loop structures, created when a single-stranded gene segment folds onto itself, initiating base-pair binding. Regions containing stem-loop structures are first transcribed into a primary miRNA (pri-miRNA), then processed by the Drosha/DCGR8 complex into 70–80 bp structures known as precursor miRNAs (pre-miRNAs). Pre-miRNAs are transported out of the nucleus into the cytoplasm, where further processing by the Dicer enzyme leaves the final mature -5p and -3p miRNAs [5–7]. Previous research suggests that only one mature miRNA from each pre-miRNA is predominantly preserved and functional, while the other is degraded [7, 8]. Despite this, there are cases in which both mature arms of a miRNA pair are maintained (e.g., miR-140-5p and miR-140-3p during chondrogenic differentiation [9]), and cases in which -5p and -3p miRNA pairs can perform synergistic regulatory roles during disease [10–12].

Exploring circulating miRNAs in OA, a miRNA-sequencing study identified a pair of miRNAs, namely miR-335-5p and miR-335-3p, to be elevated in blood plasma from individuals with early-stage radiographic knee OA compared to late-stage [13]. Subsequent bioinformatic analyses for miR-335-5p and miR-335-3p revealed unique and overlapping predicted gene targets [13]. However, since these miRNAs were identified in circulation, the relevance of miR-335-5p and miR-335-3p in local joint tissues during OA remains unknown. One study reported miR-335-5p expression is downregulated in knee OA versus normal chondrocytes [14], while conversely a separate study reported miR-335-5p is upregulated in hip OA cartilage versus healthy cartilage [15]. A third study reported miR-335-5p is downregulated in bone-marrow mesenchymal stem cells from OA versus non-OA controls [16]. Whereas these previous studies primarily focus on a single tissue or cell type, the current study aims to characterize expression patterns, validate gene targets, and assess functional effects of miR-335-5p and miR-335-3p in multiple primary human tissues from both knee and hip OA to compare and contrast their potential roles in OA pathology.

## Patients and methods

### Participants

Late-stage knee and hip OA biospecimens were procured through our Henry Ford Health OA Biobank from study participants undergoing total joint arthroplasty for moderate to severe symptomatic OA with Kellgren-Lawrence grades 3 or 4 that previously failed conservative management. Early-stage knee OA biospecimens were obtained from individuals undergoing knee arthroscopy with Kellgren-Lawrence grades 0 or 1 and mild symptoms. A detailed description of the cohorts is provided in Supplemental Table 1, and participant characteristics for each experiment are provided in Table 1. All participants provided written informed consent. The study protocol followed ethical guidelines set by the Henry Ford Health Institutional Review Board (IRB #13995).

**Table 1:** Patient characteristics by experiment

Experimental group	Late knee OA (Fig. 1C, D)	Late hip OA (Fig. 1E, F)	Early knee OA (Fig. 1A, B, G, H)	Array (Supp. Figure 1)	Validation (Fig. 2, 3, Supp. Figure 2)	Oil-Red-O (Fig. 4)
***N***	20	10	7	3	6	3
Age, mean (SD)	67.3 (10.0)	69.7 (5.9)	31.3 (10.3)	71.2 (9.4)	72.4 (4.2)	60.3 (4.7)
Sex, *N* (%) female	14 (70.0)	5 (50.0)	2 (28.6)	3 (100)	3 (50.0)	0 (0.0)
BMI, mean (SD)	32.9 (5.1)	29.0 (3.8)	28.3 (6.0)	31.7 (8.3)	35.9 (5.5)	29.0 (0.4)
Race, *N* (%) White	11 (64.7)	9 (90.0)	6 (85.7)	2 (66.7)	5 (83.3)	3 (100)

*BMI* Body mass index, *SD* Standard deviation

### Biospecimen collection and RNA isolation

Knee OA tissues included subchondral bone, articular cartilage, meniscus, infrapatellar fat pad, synovium, anterior cruciate ligament, and vastus medialis oblique muscle. Hip OA tissues included subchondral bone, articular cartilage, labrum, acetabular fossa fat, synovium, ligamentum teres, and quadratus femoris muscle. Tissues were processed and RNA was isolated following our previously published protocol [17]. Tissue explants were transfected with 100 nM miRIDIAN miR-335-5p or miR-335-3p Inhibitor or Mimic with 2.5 µl Dharmafect 1 transfection reagent (Horizon Discovery) following the manufacturer’s instructions. All tissues were cultured in serum-free DMEM with 1% penicillin–streptomycin for 24 h at 37 °C. For knee OA synovial fluid, RNA was isolated using the miRNeasy Plasma/Serum Advanced Kit (QIAGEN Inc.), following the manufacturer’s instructions.

### Bioinformatic analyses

Putative miRNA gene targets were predicted by aggregating results from the miRDIP v.5.2, miRDB, TargetScan 8.0, and DIANA-Tarbase v.8 databases, and entered into Ingenuity Pathway Analysis software (QIAGEN Inc.) for core analysis. Gibb’s free energy values (ΔG) for 70 nucleotides up- and down-stream of the miRNA seed-sequence binding sites for gene targets were calculated using UNAFold [18].

### Real-time quantitative polymerase chain reaction (RT-qPCR)

For miRNA analyses, reverse transcription was performed using the TaqMan microRNA Reverse Transcription Kit and TaqMan microRNA Assays (Applied Biosystems), following the manufacturer’s instructions. For gene analyses, cDNA was synthesized using the High-Capacity cDNA Reverse Transcription Kit (Applied Biosystems). RT-qPCR was performed for miRNAs and genes using a QuantStudio 7 Pro Real-Time PCR System (Applied Biosystems). A list of primers is provided in Supplemental Table 2. Gene expression was normalized to *GAPDH*, and miRNA expression to miR-24-3p [19]. For screening experiments, a consolidated list of putative miRNA gene targets (30 total; Supplemental Table 3) was arranged on a custom TaqMan Gene Expression Array (Applied Biosystems).

### Oil-Red-O histological analysis

Following 24 h of treatment in culture, knee OA fat pad explants were flash frozen in liquid nitrogen. Following cryosectioning, a standard Oil-Red-O staining protocol [20] was used by the Henry Ford Health Histology Core Lab. Oil-Red-O staining was quantified as percent positive stained area per total tissue section using QuPath Image Analysis software [21].

### Statistical analyses

All data analyses were performed using R 4.2.0 statistical software. We performed power analysis to determine sample size requirements to detect miRNA and gene expression differences between groups. At alpha (Type I error) of 0.05 and power of 80% (Type II error), the minimum sample size to detect an effect size of 2 was *n* = 6 per group. RT-qPCR miRNA and gene expression values were calculated via the delta-delta-Ct method [22]. Statistical significance was calculated using two-tailed Student’s t-tests and determined at *p* < 0.05.

## Results

### Characterizing expression patterns of miR-335-5p and miR-335-3p

**Fig. 1:**
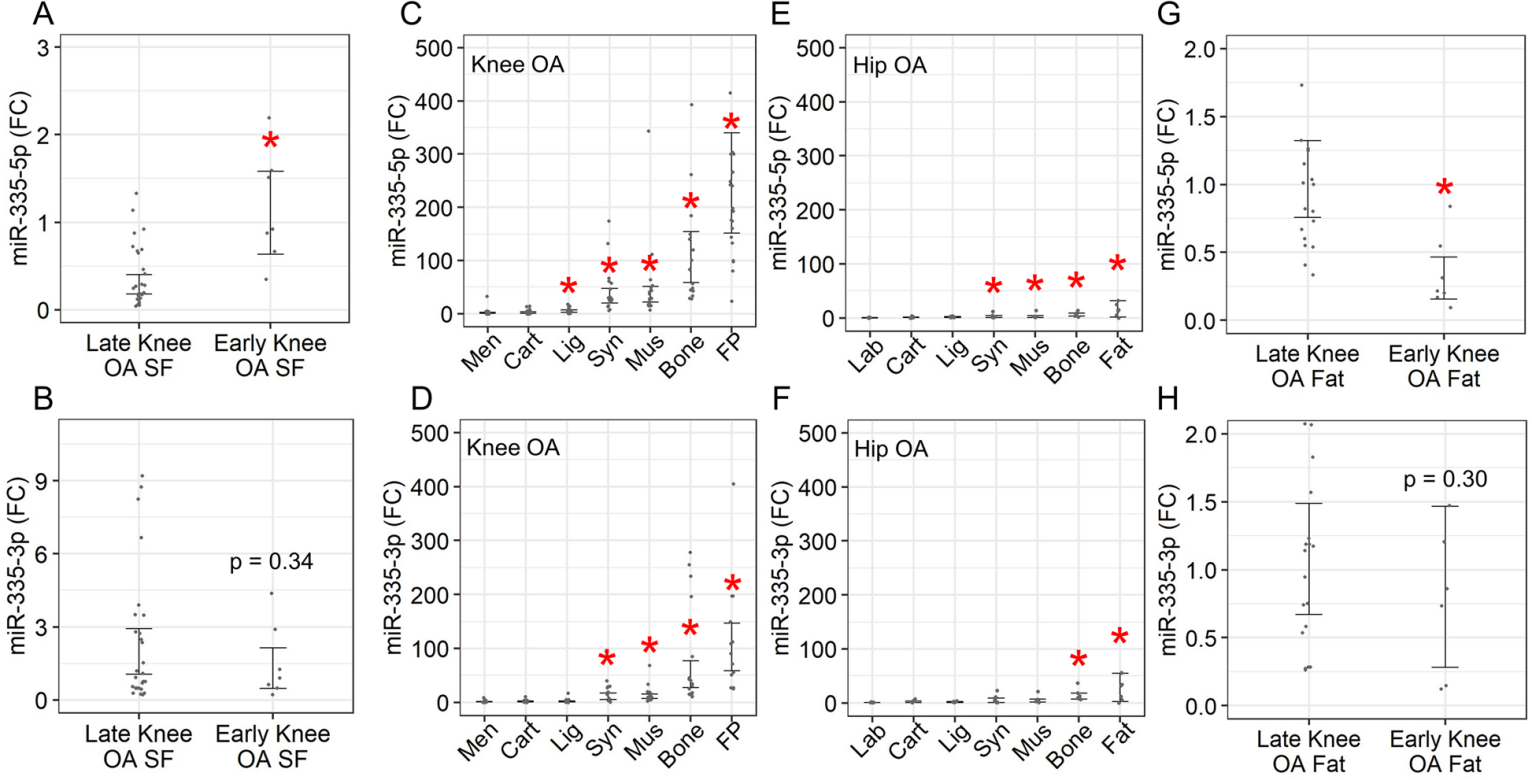
Characterizing expression patterns of miR-335-5p and miR-335-3p. **A**, **B** miRNA expression in early-stage (*n* = 7) versus late-stage (*n* = 27) knee OA synovial fluid (SF). **C**, **D** miRNA expression in matched late-stage knee OA tissues (*n* = 20; Cart, articular cartilage; Lig, anterior cruciate ligament; Syn, synovium; Mus, vastus medialis oblique muscle; FP, infrapatellar fat pad) versus meniscus (Men). **E**, **F** miRNA expression in matched late-stage hip OA tissues (*n* = 10; Cart, articular cartilage; Lig, ligamentum teres; Syn, synovium; Mus, quadratus femoris muscle) versus labrum (Lab). **G**, **H** miRNA expression in early-stage (*n* = 7) versus late-stage (*n* = 20) knee OA fat. FC, fold-change; bars = 95% CI; *p < 0.05

To expand on previous findings reporting miR-335-5p and miR-335-3p are elevated in blood plasma from early- versus late-stage radiographic knee OA [13], we measured these miRNAs in synovial fluid, a filtrate of plasma that bathes joint tissues [23]. We found higher expression of miR-335-5p in early- versus late-stage knee OA synovial fluid (Fig. 1A), but no statistically significant difference for miR-335-3p (Fig. 1B). We next measured miR-335-5p and miR-335-3p expression patterns in seven knee OA and seven hip OA tissues, all from late-stage disease. First, we found that knee OA tissues expressed both miRNAs at higher magnitudes compared to hip OA tissues (Fig. 1C–F). Second, within late-stage knee OA tissues, miR-335-5p was more abundant than miR-335-3p, with the highest expression for both miRNAs in subchondral bone and fat pad (Fig. 1C, D). Third, within late-stage hip OA tissues, miR-335-5p and miR-335-3p had comparable expression, with the highest expression for both miRNAs again in subchondral bone and acetabular fat (Fig. 1E, F). Most striking from these data, miR-335-5p showed an average 227-fold increase in late-stage knee OA fat pad compared to meniscus — the lowest expressing tissue (Fig. 1C). We therefore prioritized fat tissue for interrogation in early-stage knee OA, and found reduced levels of miR-335-5p (Fig. 1G), contrary to findings in plasma [13] and synovial fluid (Fig. 1A), and no significant differences in miR-335-3p (Fig. 1H). This led us to focus on miR-335-5p in late-stage knee OA fat pad for subsequent investigation.

### Identifying gene targets of miR-335-5p and miR-335-3p

**Fig. 2:**
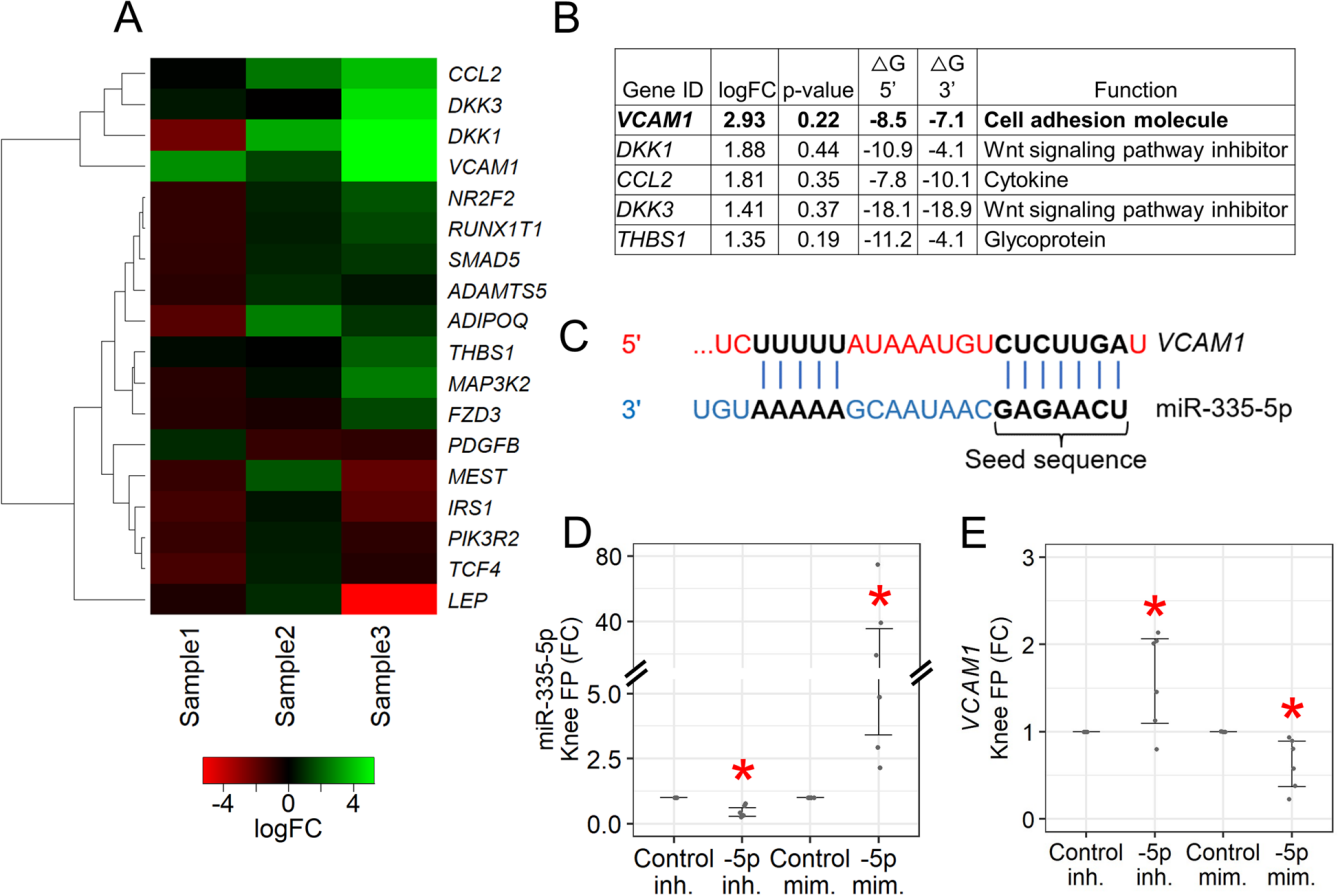
Identifying gene targets of miR-335-5p. **A** Heatmap from unsupervised clustering of miR-335-5p gene target expression changes following miR-335-5p inhibition in late-stage knee OA fat pad (*n* = 3). Two genes (*COL10A1* and *WNT10A*) with undetermined Ct values were not included. **B** Top 5 gene targets with the largest positive logFC following miR-335-5p inhibition. ΔG = Gibb’s free energy value. **C** miR-335-5p seed-sequence binding region in *VCAM1* predicted by TargetScan 8.0. **D**, **E** Expression changes in late-stage knee OA fat pad following miR-335-5p inhibitor versus control inhibitor treatment, and miR-335-5p mimic versus control mimic treatment (*n* = 6). -5p = miR-335-5p; inh. = inhibitor; mim. = mimic; FC = fold-change; bars = 95% CI; *p < 0.05

**Fig. 3:**
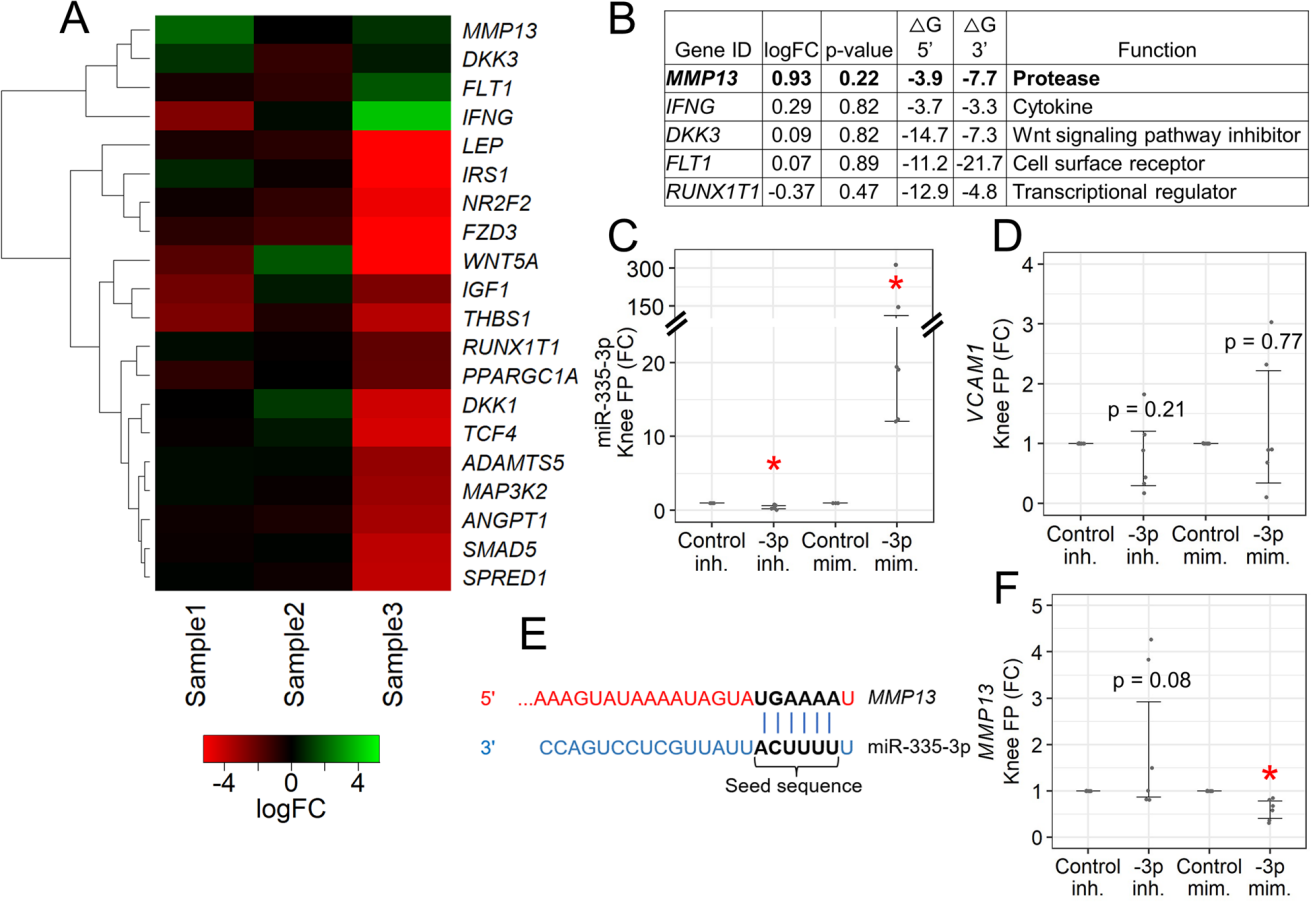
Identifying gene targets of miR-335-3p. **A** Heatmap from unsupervised clustering of miR-335-3p gene target expression changes following miR-335-3p inhibition in late-stage knee OA fat pad (*n* = 3). Two genes (*COL2A1* and *SHH*) with undetermined Ct values were not included. **B** Top 5 gene targets with largest positive logFC following miR-335-3p inhibition. ΔG = Gibb’s free energy value. **C**, **D**, and **F** Expression changes in late-stage knee OA fat pad following miR-335-3p inhibitor versus control inhibitor treatment, and miR-335-3p mimic versus control mimic treatment (*n* = 6). -3p = miR-335-3p; inh. = inhibitor; mim. = mimic; FC = fold-change; bars = 95% CI; *p < 0.05. **E** miR-335-3p seed-sequence binding region in *MMP13* predicted by TargetScan 8.0

To build on previous findings predicting 185 unique gene targets for miR-335-5p, 177 unique gene targets for miR-335-3p, and 10 overlapping gene targets for both [13] (Supplemental Table 4), we performed expanded bioinformatic analyses, identifying 4569 predicted gene targets for miR-335-5p and 7461 for miR-335-3p. These lists were refined by hand-selecting genes: (1) containing the seed-sequence binding region for either miRNA, (2) identified in the OsteoDIP database [24], or (3) reported in the literature to be involved in OA. For screening experiments, our final list consisted of 8 unique gene targets for miR-335-5p, 10 for miR-335-3p, and 12 overlapping gene targets for both (Supplemental Table 3). We measured expression of these gene targets in late-stage knee OA fat pad explants with confirmed inhibition of miR-335-5p and miR-335-3p (Supplemental Fig. 1). Hypothesizing direct gene targets would show increased expression following miRNA inhibition, we filtered for genes with the largest positive logFC (Figs. 2A and 3A). Additionally, we hypothesized that miRNAs would be more likely to bind to gene targets with higher site-accessibility, therefore we assessed the predicted ΔG of the 70-nucleotide 5′ and 3′ flanking regions of seed-sequence binding sites. Regions with ΔG values closer to 0 are considered more stable, and thus more likely to be targeted by miRNAs [18] (Figs. 2B and 3B). Based on these analyses, and its upregulation across all three samples (Fig. 2A), vascular cell adhesion molecule 1 (*VCAM1*; Fig. 2C) was prioritized for miR-335-5p validation experiments using both inhibitor and mimic treatments in independent samples (Fig. 2D). We observed the expected inverse expression pattern between *VCAM1* and miR-335-5p (Fig. 2E), but not miR-335-3p (Fig. 3C, D). Among miR-335-3p gene targets, matrix metalloproteinase 13 (*MMP13*; Fig. 3E) was prioritized, with validation experiments showing decreased expression following miR-335-3p mimic treatment, and a trend towards increased expression following inhibitor treatment (*p* = 0.08; Fig. 3F). These data suggest *VCAM1* and *MMP13* are likely gene targets of miR-335-5p and miR-335-3p, respectively, in late-stage knee OA fat pad, though further validation is required.

### Assessing functional effects of miR-335-5p in knee OA fat pad

**Fig. 4:**
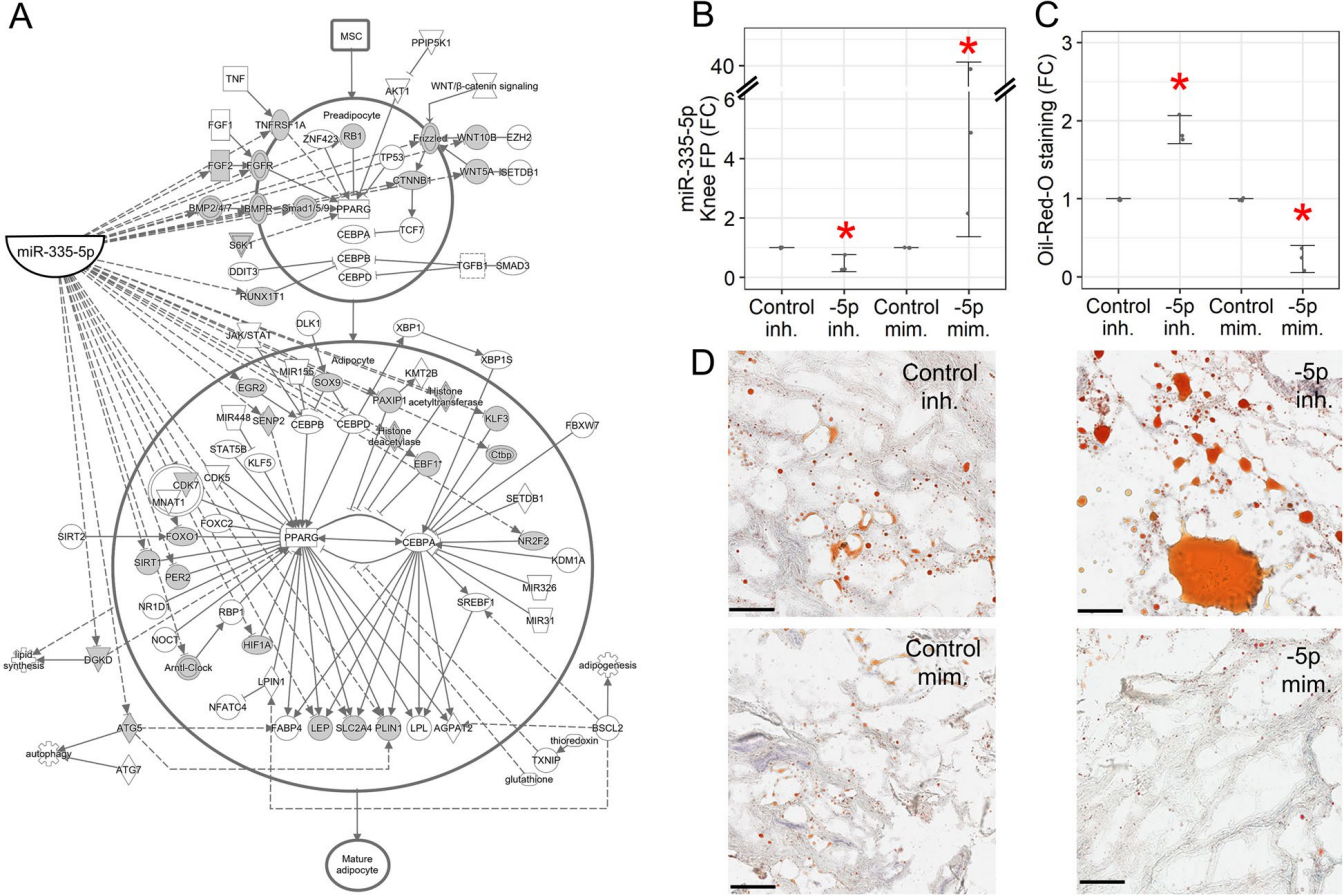
Assessing functional effects of miR-335-5p in knee OA fat pad. **A** Canonical adipogenesis network generated from Ingenuity Pathway Analysis software, shaded symbols = predicted miR-335-5p targets. **B** miRNA expression changes following miR-335-5p inhibitor versus control inhibitor treatment and miR-335-5p mimic versus control mimic treatment in late-stage knee OA fat pad explants used for Oil-Red-O staining (*n* = 3). **C** Quantification of Oil-Red-O staining in miR-335-5p inhibitor or mimic treated explants relative to control inhibitor or mimic, respectively (*n* = 3). -5p = miR-335-5p; inh. = inhibitor; mim. = mimic; FC, fold-change; bars = 95% CI; *p < 0.05. **D** Representative images of late-stage knee OA fat pad sections corresponding to each group in panel **C**; red = Oil-Red-O stain, magnification = 10 × , scale bar = 100 µm

Since miR-335-5p modulation induced stronger gene expression changes (Fig. 2B) than did miR-335-3p (Fig. 3B), we sought to determine the effect of miR-335-5p on biological pathways that might be relevant in knee OA fat pad. Utilizing the 4569 predicted miR-335-5p gene targets described above, we performed pathway analysis and found 44 gene targets to be enriched among 135 total molecules in a canonical adipogenesis network (*p* = 2.1e − 5; Fig. 4A). To assess the net functional effect of this, we modulated miR-335-5p in late-stage knee OA fat pad (Fig. 4B) and performed Oil-Red-O staining. We observed more lipids following miR-335-5p inhibitor treatment and less following mimic treatment (Fig. 4C, D). To elucidate potential mechanisms underlying this observation, we modulated miR-335-5p in independent knee OA fat pad explants (Fig. 2D) and measured four genes from the canonical adipogenesis network — two master regulators without the miR-335-5p seed-sequence binding site (*PPAR*γ and *CEBPA*) and two putative direct gene targets with the site (*LEP* and *DGKD*). Following mimic treatment, we found increased expression of *PPAR*γ and a trend towards increased expression of *LEP* (*p* = 0.08; Supplemental Fig. 2), but these findings are inconsistent with the reduction in lipids we observed (Fig. 4C, D), and do not follow the inverse expression pattern expected of direct gene targets of miRNAs. Though the mechanism remains unclear, our data suggest the net effect of miR-335-5p in late-stage knee OA fat pad is to reduce lipid content.

## Discussion

This study is the first to directly compare and contrast the roles of a miRNA pair in multiple primary human joint tissues from both knee and hip OA. Our miRNA expression data identified late-stage knee OA fat pad as a tissue of interest in which *VCAM1* and *MMP13* are likely direct gene targets of miR-335-5p and miR-335-3p, respectively. We also show functional effects of miR-335-5p on modulating total lipid content in fat pad. Taken together our findings suggest that miR-335-5p and miR-335-3p expression patterns, gene targets, and functional effects may depend on the tissue, joint, and stage of OA. This builds our understanding of miRNA mechanisms in OA, suggesting that -5p and -3p arms of a miRNA function in a highly context-dependent manner and should not be considered the same miRNA.

Despite considerable differences in magnitudes, bone and fat exhibited the highest expression of miR-335-5p and miR-335-3p across tissues and joints; similarly, meniscus/labrum and cartilage exhibited the lowest expression. Since these two miRNAs were initially identified in circulation during OA [13], it is not surprising that they are more abundant in vascular versus avascular joint tissues. Expression in bone is also expected given previous reports of miR-335-5p regulating bone homeostasis in OA [16]. However, expression in fat tissues may have been missed in studies focused only on the classical OA tissues such as cartilage and synovium [14, 15]. Links between miR-335-5p and adipogenesis have previously been reported in acute coronary syndrome [25], reinforcing our finding of its role in regulating lipid content in knee OA fat pad. Though it remains unknown from our data whether miR-335-5p is regulating de novo adipogenesis or merely lipid accumulation, overall in OA pathology, excess adipose tissue is detrimental [26]. The decrease in lipid content we observed following miR-335-5p mimic treatment suggests it could play a role in mitigating OA through lipid reduction, and the increases in key adipogenesis regulators we observed could be a compensatory mechanism to increase lipid production in the context of miR-335-5p reducing total lipids. In sum, these findings highlight a tissue-specific role for miR-335-5p and the need to consider tissue type when elucidating the function of miRNAs.

Since miR-335-5p exhibited higher abundance across knee OA tissues than miR-335-3p, miR-335-5p may be the preferentially preserved arm of the pair; however, there was no such distinction between these arms across hip OA tissues. We postulate that miR-335-5p may function in a site-specific manner that is more prominent in knee OA than hip OA, and further, to late-stage knee OA tissues than early-stage. One explanation to reconcile the discrepant findings between plasma and synovial fluid (where miR-335-5p is elevated in early-stage knee OA) and fat pad (where miR-335-5p is elevated in late-stage knee OA) is that circulating miR-335-5p during early-stage knee OA becomes sequestered in local joint tissues during later stages of disease to regulate gene target expression. Through downregulation of *VCAM1* and *MMP13*, our data suggest miR-335-5p and miR-335-3p may both be functioning in a protective manner to mitigate common pathological processes observed in OA, such as inflammation and matrix degradation, respectively. Our findings suggest that this could be a novel synergistic disease mechanism driven by a miRNA pair that warrants further investigation.

Several limitations may influence the interpretation of our results. Since this study builds upon a previous study comparing early- to late-stage knee OA [13], a key limitation is the lack of a healthy control cohort with which to compare our miR-335-5p and miR-335-3p expression data. Second, our custom screening array consisted of relatively few genes (30 total) and few samples (3 total) and therefore limited our potential to discover novel targets of this miRNA pair. A larger sample size and high-throughput techniques such as RNA-sequencing would provide more comprehensive profiling of gene expression changes across the transcriptome following miRNA modulation. Third, as we did not perform reporter construct assays to validate the direct regulation of miR-335-5p or miR-335-3p to *VCAM1* or *MMP13*, respectively, we cannot report that these are validated gene targets. Finally, our investigation into mechanisms underlying miR-335-5p regulation of lipid content was confined to four candidate genes identified from the canonical adipogenesis network. As we were unable to resolve the direct mechanism in the current study, experiments are still needed to explore other gene targets governing lipid content, and the overall impact of miR-335-5p regulation of lipid content on OA outcomes.

## Conclusion

In this report, we show miR-335-5p is highly expressed in late-stage knee OA fat pad, induces changes to *VCAM1* expression, and functions to reduce lipid content in fat pad. We also show miR-335-3p downregulates *MMP13* expression, suggesting that this miRNA pair may function in parallel to mitigate common pathological processes during OA. In addition to providing insight into tissue-, joint-, and stage-specific mechanisms underlying OA pathology, our study also informs miRNA biology by demonstrating the complexity with which arms of a miRNA pair may function.

## Supplementary Information

**Additional file 1: Supplemental Figure 1.** A) miR-335-5p and B) miR-335-3p inhibition in late-stage knee OA fat pad explants used for custom TaqMan Gene Expression Array. *n*=3; -5p = miR-335-5p; -3p = miR-335-3p; inh. = inhibitor; *p < 0.05 versus control inhibitor.

**Additional file 2: Supplemental Figure 2.** Gene expression changes in key adipogenesis pathway components following miR-335-5p modulation. A) *PPARγ* = peroxisome proliferator activated receptor gamma. B) *CEBPA* = CCAAT enhancer binding protein alpha. C) *LEP* = leptin. D) *DGKD* = diacylglycerol kinase delta. *n*=6; -5p = miR-335-5p; inh. = inhibitor; mim. = mimic; FC = fold-change; bars = 95% CI; *p < 0.05 versus control inhibitor or mimic.

**Additional file 3: Supplemental Table 1.** Detailed description of OA cohorts.

**Additional file 4: Supplemental Table 2.** List of real-time quantitative polymerase chain reaction primers.

**Additional file 5: Supplemental Table 3.** Filtered list of miR-335-5p and miR-335-3p gene targets used in custom TaqMan Gene Expression Array.

**Additional file 6: Supplemental Table 4.** Lists of unique and overlapping miR-335-5p and miR-335-3p gene targets reported by Ali et al. *Osteoarthritis and Cartilage* (2020).

## Data Availability

All datasets used in this publication are available from the corresponding author upon request.

## Abbreviations

OA: Osteoarthritis
miRNA: MicroRNA
pri-miRNA: Primary microRNA
pre-miRNA: Precursor microRNA
*VCAM1*: Vascular cell adhesion molecule 1
*MMP13*: Matrix metalloproteinase 13
*PPAR*γ: Peroxisome proliferator activated receptor gamma
*CEBPA*: CCAAT enhancer binding protein alpha
*LEP*: Leptin
*DGKD*: Diacylglycerol kinase delta
