# Supplementary figures and images for: Characterization of miR-335-5p and miR-335-3p in human osteoarthritic tissues
Source: Arthritis Res Ther. 2023 Jun 16;25:105. doi: 10.1186/s13075-023-03088-6 (PMC10273720; doi:10.1186/s13075-023-03088-6)

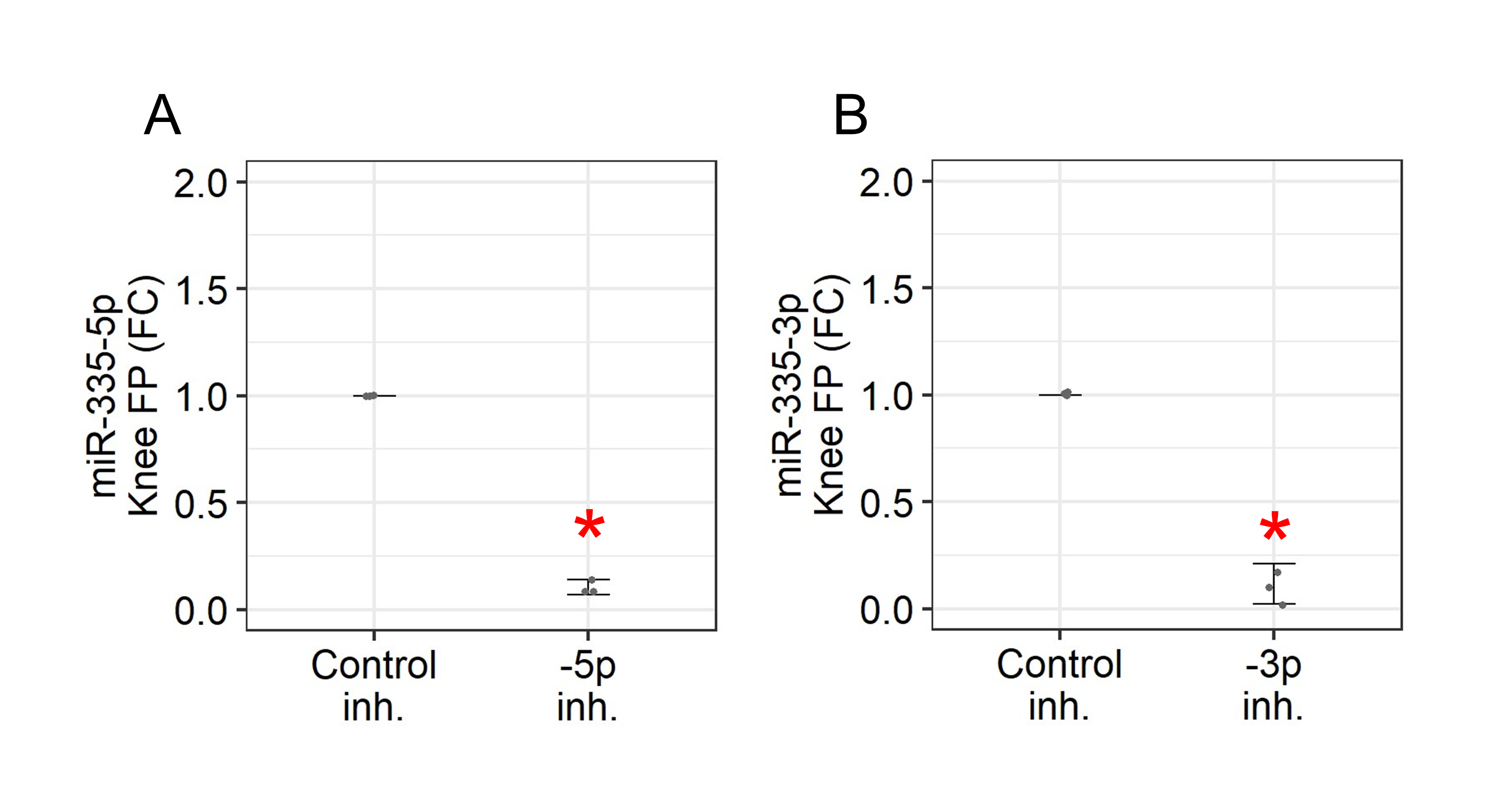

Supplement: Supplementary file 1 — Additional file 1: Supplemental Figure 1. A) miR-335-5p and B) miR-335-3p inhibition in late-stage knee OA fat pad explants used for custom TaqMan Gene Expression Array. n=3; -5p = miR-335-5p; -3p = miR-335-3p; inh. = inhibitor; *p < 0.05 versus control inhibitor. [file 13075_2023_3088_MOESM1_ESM.tiff]

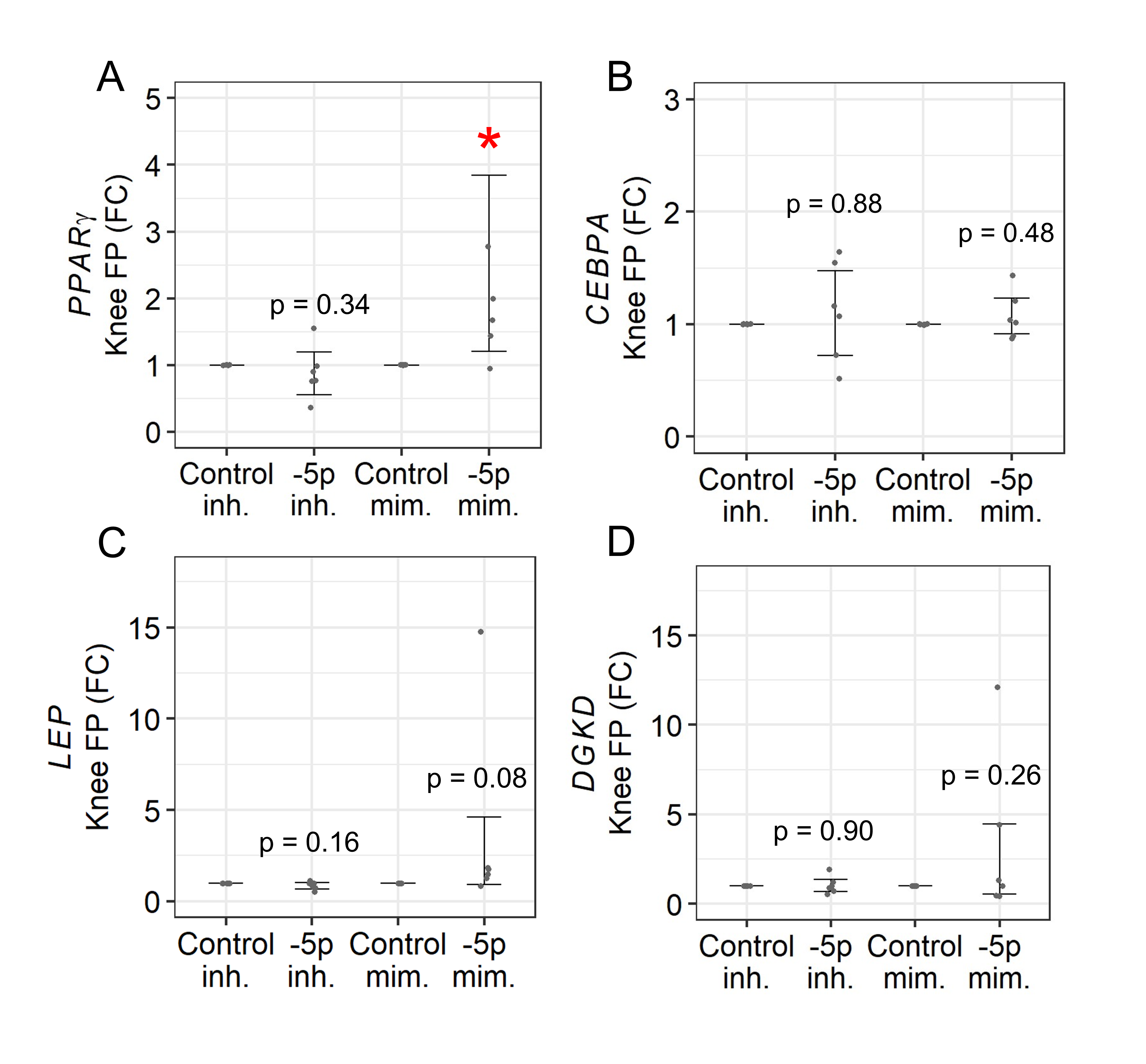

Supplement: Supplementary file 2 — Additional file 2: Supplemental Figure 2. Gene expression changes in key adipogenesis pathway components following miR-335-5p modulation. A) PPARγ = peroxisome proliferator activated receptor gamma. B) CEBPA = CCAAT enhancer binding protein alpha. C) LEP = leptin. D) DGKD = diacylglycerol kinase delta. n=6; -5p = miR-335-5p; inh. = inhibitor; mim. = mimic; FC = fold-change; bars = 95% CI; *p < 0.05 versus control inhibitor or mimic. [file 13075_2023_3088_MOESM2_ESM.tiff]
