# Supplementary material for: Characterization of miR-335-5p and miR-335-3p in human osteoarthritic tissues
Source: Arthritis Res Ther. 2023 Jun 16;25:105. doi: 10.1186/s13075-023-03088-6 (PMC10273720; doi:10.1186/s13075-023-03088-6)
Supplement: Supplementary file 3 — Additional file 3: Supplemental Table 1. Detailed description of OA cohorts. [file 13075_2023_3088_MOESM3_ESM.docx]

|  | |  |  |  |  |
| --- | --- | --- | --- | --- | --- |
| **Cohort** | **KL Grades** | | **Surgery Type** | **Patient-reported OA symptoms** | **Failed conservative management?** |
| Early-stage knee OA | 0,1 | | Knee arthroscopy | Mild | No |
| Late-stage knee OA | 3,4 | | Total knee arthroplasty | Moderate to Severe | Yes |
| Late-stage hip OA | 3,4 | | Total hip arthroplasty | Moderate to Severe | Yes |
