# Supplementary material for: Characterization of miR-335-5p and miR-335-3p in human osteoarthritic tissues
Source: Arthritis Res Ther. 2023 Jun 16;25:105. doi: 10.1186/s13075-023-03088-6 (PMC10273720; doi:10.1186/s13075-023-03088-6)
Supplement: Supplementary file 4 — Additional file 4: Supplemental Table 2. List of real-time quantitative polymerase chain reaction primers. [file 13075_2023_3088_MOESM4_ESM.docx]

| **miRNA/Gene ID** | **Assay ID (TaqMan) or 5’–3’ Sequences (SYBR)** |
| --- | --- |
| hsa-miR-335-5p | 000546 |
| hsa-miR-335-3p | 002185 |
| hsa-miR-24-3p (reference miRNA) | 000402 |
| *GAPDH* (TaqMan reference gene) | Hs99999905_m1 |
| *VCAM1* | Hs01003372_m1 |
| *GAPDH* (SYBR reference gene) | CATCACTGCCACCCAGAAGACTG – forward  ATGCCAGTGAGCTTCCCGTTCAG – reverse |
| *MMP13* | CCTTGATGCCATTACCAGTCTCC – forward  AAACAGCTCCGCATCAACCTGC – reverse |
| *LEP* | GCTGTGCCCATCCAAAAAGTCC – forward  CCCAGGAATGAAGTCCAAACCG – reverse |
| *DGKD* | TCACCGAAGACTTCAGCGAGGA – forward  AGGAGATGACCACCGAGTGCTG – reverse |
| *PPARγ* | AGCCTGCGAAAGCCTTTTGGTG – forward  GGCTTCACATTCAGCAAACCTGG – reverse |
| *CEBPA* | TGGACAAGAACAGCAACGAG – forward  TTGTCACTGGTCAGCTCCAG – reverse |
