# Supplementary material for: Characterization of miR-335-5p and miR-335-3p in human osteoarthritic tissues
Source: Arthritis Res Ther. 2023 Jun 16;25:105. doi: 10.1186/s13075-023-03088-6 (PMC10273720; doi:10.1186/s13075-023-03088-6)
Supplement: Supplementary file 5 — Additional file 5: Supplemental Table 3. Filtered list of miR-335-5p and miR-335-3p gene targets used in custom TaqMan Gene Expression Array. [file 13075_2023_3088_MOESM5_ESM.docx]

|  | |  | | |  | |  |
| --- | --- | --- | --- | --- | --- | --- | --- |
| Gene | Seed-sequences | | OsteoDIP? | Related OA references | | Primary reasons for inclusion | |
| *ADAMTS5* | 5p,3p | | Yes | PMID: 15800624 | | Seed sequence for both -5p and -3p | |
| *ADIPOQ* | 5p | | Yes | PMID: 30115130 | | adipogenesis | |
| *ANGPT1* | 3p | | Yes | PMID: 22641138 | | angiogenesis | |
| *CCL2* | 5p | | Yes | PMID: 27746376 | | inflammation | |
| *COL2A1* | 3p | | Yes | PMID: 16755660 | | anabolism | |
| *COL10A1* | 5p | | Yes | PMID: 20959023 | | chondrocyte hypertrophy | |
| *DKK1* | 5p,3p | | Yes | PMID: 25080367 | | Seed sequence for both -5p and -3p | |
| *DKK3* | 5p,3p | | Yes | PMID: 26687825 | | Seed sequence for both -5p and -3p | |
| *FLT1* | 3p | | Yes | PMID: 30873504 | | angiogenesis | |
| *FZD3* | 5p,3p | | Yes | PMID: 27146333 | | Seed sequence for both -5p and -3p | |
| *IFNG* | 3p | | Yes | PMID: 36105611 | | inflammation | |
| *IGF1* | 3p | | Yes | PMID: 34717735 | | proliferation | |
| *IRS1* | 5p,3p | | Yes | PMID: 17129375 | | Seed sequence for both -5p and -3p | |
| *LEP* | 5p,3p | | Yes | PMID: 29620639 | | Seed sequence for both -5p and -3p | |
| *MAP3K2* | 5p,3p | | No | PMID: 18698181 | | Seed sequence for both -5p and -3p | |
| *MMP13* | 3p | | Yes | PMID: 23298463 | | catabolism | |
| *MEST* | 5p | | Yes | PMID: 26243143 | | mir335 host gene | |
| *NR2F2* | 5p,3p | | Yes | PMID: 27345768 | | Seed sequence for both -5p and -3p | |
| *PDGFB* | 5p | | No | PMID: 32208385 | | angiogenesis | |
| *PIK3R2* | 5p | | No | PMID: 31886203 | | PI3K signaling pathway | |
| *PPARGC1A* | 3p | | Yes | PMID: 25940958 | | mitochondrial biogenesis | |
| *RUNX1T1* | 5p,3p | | Yes | PMID: 24361742 | | Seed sequence for both -5p and -3p | |
| *SHH* | 3p | | No | PMID: 19915594 | | Hedgehog signaling pathway | |
| *SMAD5* | 5p,3p | | No | PMID: 25489490 | | Seed sequence for both -5p and -3p | |
| *SPRED1* | 3p | | Yes | PMID: 22641138 | | angiogenesis | |
| *TCF4* | 5p,3p | | Yes | PMID: 23603903 | | Seed sequence for both -5p and -3p | |
| *THBS1* | 5p,3p | | Yes | PMID: 29238343 | | Seed sequence for both -5p and -3p | |
| *VCAM1* | 5p | | Yes | PMID: 19644856 | | inflammation | |
| *WNT5A* | 3p | | Yes | PMID: 31964233 | | Wnt signaling pathway | |
| *WNT10A* | 5p | | Yes | PMID: 31964233 | | Wnt signaling pathway | |
