# Supplementary material for: Characterization of miR-335-5p and miR-335-3p in human osteoarthritic tissues
Source: Arthritis Res Ther. 2023 Jun 16;25:105. doi: 10.1186/s13075-023-03088-6 (PMC10273720; doi:10.1186/s13075-023-03088-6)
Supplement: Supplementary file 6 — Additional file 6: Supplemental Table 4. Lists of unique and overlapping miR-335-5p and miR-335-3p gene targets reported by Ali et al. Osteoarthritis and Cartilage (2020). [file 13075_2023_3088_MOESM6_ESM.docx]

|  |  |  |
| --- | --- | --- |
|  |  |  |
| **Unique miR-335-5p targets**  **N=185** | **Unique miR-335-3p targets**  **N=177** | **Overlapping targets**  **N=10** |
| *ADCY3* | *ACBD5* | *CTDSPL2* |
| *ADGRL2* | *ACSL6* | *ETF1* |
| *AHSG* | *AEBP2* | *GALNT1* |
| *AP3B1* | *AFF2* | *HNRNPA3* |
| *AP3S1* | *AFF4* | *MAP3K2* |
| *APTX* | *AGFG1* | *SH3KBP1* |
| *ARF4* | *ANK1* | *STIM2* |
| *ARGLU1* | *ANKRD12* | *TCF4* |
| *ARHGAP18* | *ARFGEF2* | *UBE4A* |
| *ARPC5L* | *ARID4B* | *ZBTB10* |
| *ASB7* | *ATP2A2* |  |
| *ATP1B1* | *ATP5G3* |  |
| *B3GNT5* | *BRCC3* |  |
| *BCL2L2* | *BRD1* |  |
| *BSN* | *BRWD1* |  |
| *CALU* | *C1GALT1* |  |
| *CASP7* | *CALM1* |  |
| *CCNT2* | *CCDC170* |  |
| *CD164* | *CCDC88A* |  |
| *CDC7* | *CCPG1* |  |
| *CDK2* | *CD47* |  |
| *CEP350* | *CDC73* |  |
| *CHFR* | *CDKN1B* |  |
| *CNOT7* | *CHD9* |  |
| *CRIM1* | *CITED2* |  |
| *CRKL* | *CLINT1* |  |
| *CRNKL1* | *CNOT2* |  |
| *DAAM1* | *CPEB4* |  |
| *DCAF10* | *CPNE3* |  |
| *DCX* | *CREBZF* |  |
| *DOCK4* | *CRIPT* |  |
| *DOCK5* | *CRISPLD1* |  |
| *EEF2K* | *CUL3* |  |
| *EFNB1* | *CUL5* |  |
| *EIF4A2* | *DCUN1D1* |  |
| *EIF5A2* | *DYRK1A* |  |
| *ENPEP* | *EDNRA* |  |
| *EPB41L4B* | *EIF4E3* |  |
| *ESR1* | *ELK3* |  |
| *ETNK1* | *ELMO1* |  |
| *EVI5* | *EML4* |  |
| *F13A1* | *EPHA3* |  |
| *FAM107B* | *EPHA4* |  |
| *FAM131B* | *EPHA7* |  |
| *FBXO28* | *ERC2* |  |
| *FKBP1B* | *ERP44* |  |
| *FMR1* | *ETS1* |  |
| *GATA2* | *EYA4* |  |
| *GDE1* | *FAM126A* |  |
| *GJA5* | *FAM126B* |  |
| *GLYR1* | *FAM49A* |  |
| *GOSR1* | *FAT3* |  |
| *GRIA2* | *FLRT3* |  |
| *HAND1* | *FNDC3A* |  |
| *HIF1AN* | *FOS* |  |
| *HLF* | *FPGT* |  |
| *HMGCR* | *FRS2* |  |
| *HNRNPC* | *GABRB2* |  |
| *HNRNPR* | *GATAD2B* |  |
| *HOXD8* | *GPR180* |  |
| *IGF2BP2* | *GPR85* |  |
| *IL17RD* | *GRIK2* |  |
| *ILF3* | *GSPT1* |  |
| *ISL1* | *GXYLT1* |  |
| *JAG1* | *HLCS* |  |
| *JMJD1C* | *HMGA2* |  |
| *KAT7* | *HTR2C* |  |
| *KDM2B* | *ID4* |  |
| *KDM4C* | *IGF1R* |  |
| *KDSR* | *INSIG1* |  |
| *KLHL15* | *JCAD* |  |
| *KLHL28* | *JMY* |  |
| *KMT5A* | *KCNMA1* |  |
| *KPNA1* | *KCNQ5* |  |
| *KPNA3* | *KITLG* |  |
| *KPNA6* | *KLHL9* |  |
| *KRT24* | *KPNA4* |  |
| *LMX1A* | *LARP4B* |  |
| *LONRF1* | *LMO4* |  |
| *M6PR* | *LPGAT1* |  |
| *MAPK10* | *LRP2* |  |
| *MAT2B* | *LRRK2* |  |
| *MAX* | *MAGI1* |  |
| *MED21* | *MBNL1* |  |
| *MED6* | *MED1* |  |
| *MEF2D* | *MEGF10* |  |
| *MET* | *MINDY2* |  |
| *MLX* | *MKL2* |  |
| *MON2* | *MLLT3* |  |
| *MOSPD1* | *MMP16* |  |
| *MSMO1* | *MYCN* |  |
| *MTMR4* | *NAALADL2* |  |
| *MTMR9* | *NAPEPLD* |  |
| *MYBL1* | *NCKAP1* |  |
| *NAA25* | *NECAB1* |  |
| *NAA50* | *NEUROG1* |  |
| *NAP1L1* | *NFIA* |  |
| *NCKAP5* | *NFIB* |  |
| *NDFIP1* | *NOL4* |  |
| *NEBL* | *NPAS3* |  |
| *NEMP1* | *NR2F2* |  |
| *NETO2* | *NR3C1* |  |
| *NFYB* | *NRIP1* |  |
| *NIPA2* | *NSD2* |  |
| *NOTCH2* | *OGN* |  |
| *NPAS4* | *OTUD4* |  |
| *NR4A3* | *PARVA* |  |
| *NRXN1* | *PAX6* |  |
| *NXPE3* | *PAX8* |  |
| *OSBPL3* | *PCGF5* |  |
| *PCDH9* | *PCMTD1* |  |
| *PCNX1* | *PDIK1L* |  |
| *PLPP3* | *PDLIM5* |  |
| *POU2F3* | *PDS5B* |  |
| *POU5F1* | *PGAP1* |  |
| *PPM1E* | *PGM2L1* |  |
| *PPP1R3A* | *PHACTR2* |  |
| *PPP6C* | *PLCB4* |  |
| *PRDM10* | *PMEPA1* |  |
| *PRDM2* | *PPP3CA* |  |
| *PRKAA2* | *PRKG1* |  |
| *PRPF38B* | *PRLR* |  |
| *PRR5L* | *PRPF40A* |  |
| *PSD3* | *PRRX1* |  |
| *PUM1* | *PTPRD* |  |
| *PYGO2* | *PUM2* |  |
| *RAB14* | *RAD23B* |  |
| *RAD9B* | *RC3H1* |  |
| *RAP1A* | *RORA* |  |
| *RASA1* | *RP2* |  |
| *RB1* | *RPGRIP1L* |  |
| *RBFOX2* | *RPS6KA3* |  |
| *RBM12* | *RTN3* |  |
| *RBM7* | *RUNX1T1* |  |
| *RBMX* | *RYR2* |  |
| *RETREG3* | *SAMD8* |  |
| *RGS7BP* | *SATB1* |  |
| *RNF141* | *SATB2* |  |
| *ROCK1* | *SEMA3A* |  |
| *RPRM* | *SEMA3D* |  |
| *RSBN1* | *SENP6* |  |
| *RSPO2* | *SHOX2* |  |
| *RSRC2* | *SIX4* |  |
| *RYBP* | *SKIL* |  |
| *SALL1* | *SLC17A6* |  |
| *SCAPER* | *SLC25A13* |  |
| *SCN2A* | *SLC25A24* |  |
| *SECISBP2L* | *SLC26A7* |  |
| *SEPHS1* | *SLC9A2* |  |
| *SHANK2* | *SMAD5* |  |
| *SLC25A44* | *SOS1* |  |
| *SLC7A11* | *SOX11* |  |
| *SMARCA2* | *SPARC* |  |
| *SMG1* | *SRPK2* |  |
| *SNIP1* | *STC1* |  |
| *SORCS1* | *STXBP5* |  |
| *SP1* | *SWAP70* |  |
| *SPATA2* | *SYT14* |  |
| *SPTSSA* | *TBL1XR1* |  |
| *SREK1IP1* | *TEAD1* |  |
| *STARD7* | *TENM1* |  |
| *STRN3* | *TMEM106B* |  |
| *SYN2* | *USP15* |  |
| *THOC7* | *VAPB* |  |
| *TIAM1* | *XIRP2* |  |
| *TPR* | *YAF2* |  |
| *TPST1* | *YTHDF2* |  |
| *TTK* | *ZFP36L2* |  |
| *UBE2G1* | *ZFX* |  |
| *UBE2H* | *ZIC3* |  |
| *VAPA* | *ZMYM4* |  |
| *WAPL* | *ZNF197* |  |
| *WWP1* | *ZNF23* |  |
| *ZC4H2* | *ZNF430* |  |
| *ZDHHC2* | *ZNF449* |  |
| *ZDHHC8* | *ZNF655* |  |
| *ZHX1* | *ZNF681* |  |
| *ZKSCAN8* |  |  |
| *ZMPSTE24* |  |  |
| *ZNF532* |  |  |
| *ZNF592* |  |  |
| *ZNF621* |  |  |
| *ZNF654* |  |  |
| *ZNF800* |  |  |
| *ZRANB1* |  |  |
